# Acute Haematogenous Metacarpal Osteomyelitis in Children: A Case Report and Review of Literature

**DOI:** 10.1155/2011/674820

**Published:** 2011-08-11

**Authors:** Jordi Colomina Morales, Laura Soria Villegas, Joan Carles Monllau García

**Affiliations:** Department of Orthopaedic Surgery, Hospital de la Santa Creu i Sant Pau, Universitat Autònoma de Barcelona, 08025 Barcelona, Spain

## Abstract

Metatarsal osteomyelitis in children is a very uncommon disease, and this can make diagnosis difficult leading to a delay in treatment that can cause complications like brachymetacarpia or loss of function. We present an infant affected by granulomatous chronic disease with acute haematogenous osteomyelitis of the fourth metacarpal. *Serratia marcescens* was found to be the pathogenic agent. Treatment was antibiotics and debridement. Bone healed with little length discrepancy but functional result was normal.

## 1. Background

The most common location of acute haematogenous osteomyelitis is the metaphyseal region of long bones, with the lower extremities more commonly affected than the upper extremity [1]. In this later location, osteomyelitis of the metacarpals is even more uncommon, and few papers have been published about this condition [2–5].

The purpose of this paper is to present a case of acute haematogenous metacarpal osteomyelitis in an infant lately diagnosed of chronic granulomatous disease (CGD). To our knowledge, no previous reports have been published.

## 2. Case Report

 A 22-month-old male infant was admitted at the emergency department with pain and swelling in dorsoulnar aspect of the right hand started few hours before.

 Physical examination revealed a tender swelling over the forth metacarpal, with limited movement of both carpometacarpal and the metacarpophalangeal joints; any sign of injury, trauma, or wound was appreciated. Temperature was normal in the first examination, but at three hours after admission fever >38°C appeared. Laboratory studies showed elevation of leukocyte count (17.0 × 10^9^/L) and C-reactive protein (56.3 mg/L).

**Figure 1:**
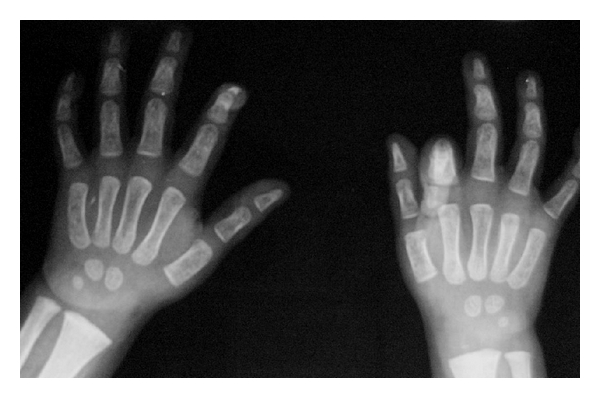
Plain radiographs showing little cortical hyperdensity of right hand fourthmetacarpal.

**Figure 2:**
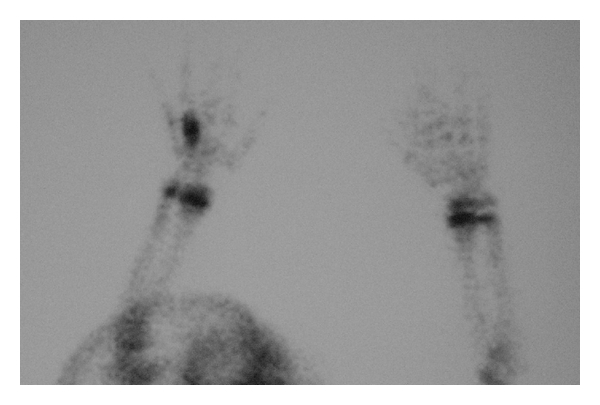
Scintigraphy showing 4th metacarpal and surrounding soft tissue hypercaptation.

**Figure 3:**
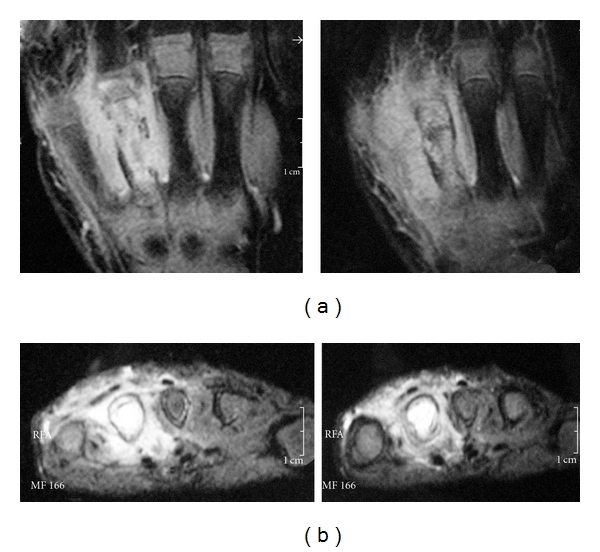
MRI findings, diaphyseal and physeal destruction, and bone oedema and soft tissue oedema.

Plain radiographs showed hyperdensity of the forth metacarpal (Figure 1). A radionuclide ^99m^Tc bone scan showed increased uptake in the forth metacarpal (Figure 2), whereas magnetic resonance images (M.R.I.) showed cortical destruction, diaphyseal and physeal involvement, and adjacent soft tissue oedema (Figure 3), all this consistent with osteomyelitis. 

**Figure 4:**
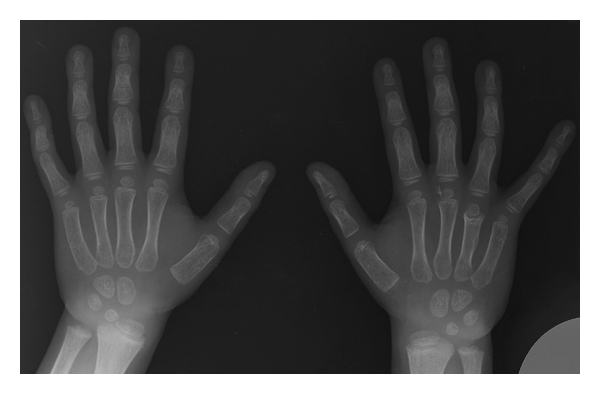
3-year posttreatment radiographs showing discrete shortening of the right hand fourth metacarpal.

 Intravenous (iv.) empirical antibiotic therapy was started with cloxacillin and gentamicin, but clinical evolution was unfair with persistent fever. Due to it, surgery was planned. Debridement and curettage were performed. Cultures were positive for *Serratia marcescens,* and antibiotic therapy with iv. cefotaxime was established. Marked clinical improvement was noted in the first week, the reason by which he returned to home with oral treatment with cefuroxime and Trimethoprim-Sulfametoxazole. The pain and swelling of the hand resolved completely 1 month after surgical debridement. In review at three years later, radiological examination showed a discrete shortening of forth metacarpal, probably caused by the physeal spread of infection, but with complete function of the hand (Figure 4). 

The patient needed two further hospital admissions due to systemic infections (one for an endocarditic infection). The uncommon presentation of osteomyelitis and other infectious episodes led the medical team to perform an immunological study, and chronic granulomatous disease (syndrome of phagocyte oxidase deficiency) was finally diagnosed (hereditary disease that causes severe and recurrent infections in children) [6, 7].

## 3. Discussion

 Metacarpal osteomyelitis in children is a very infrequent entity, and few papers with five cases have been published about this condition (Table 1) [2–5]. Of these cases, the mechanism of infection was dog scratch [2], local dissemination due to closed trauma [4], cellulitis of the hand [5], and haematogenous dissemination [3]. Our patient constitutes another case of haematogenous dissemination, but associated with CGD, that is, an hereditary immunodeficiency syndrome characterized clinically by severe recurrent bacterial and fungal infections that are difficult to treat by conventional means [6–8]. This disease is usually recognized in young children below the age of 2 years, as in our patient. 

 Early diagnosis and treatment of the osteomyelitis hold the key to a good result. According to Sonnen and Henry [9], the diagnosis of osteomyelitis required 2 of the following diagnostic criteria: purulence of the bone; a positive bone or blood culture; localized erythema, oedema, or both; a positive imaging study, either on radiography, scintigraphy, or M.R.I. Our patient showed all these criteria. It is interesting to notice the difference between the radiologic findings with mild abnormalities and the M.R.I. showing cortical destruction, diaphyseal and physeal involvement, and adjacent soft tissue oedema. 

In the setting of CGD, lower extremities seem to be the more common localization, followed by chest wall and then upper extremities [8] as it was in our case.

 As far as the bacterial pathogen, the organisms involved in the previous cases of metacarpal osteomyelitis were *Bartonella henselae*, *Coccidioides immitis, Staphylococcus aureus *[4, 5], *and Group A Beta Haemolytic-Streptococci *[5]. In our case, the microorganism was *Serratia marcescens*, an aerobic Gram-negative opportunist bacillus which can be present in the infections of chronic pathology, as the CGD [7, 10]. 

 Finally, and as far as the treatment, all cases did well at the end of followup. In our case, the result was a discrete shortening of the metacarpal but without clinical involvement. In this regard, routine surgical exploration of an osteomyelitic focus is not recommended, but failure to respond to antibiotics or evidence of local abscess formation should be the main indications, as it happened in our case.

##  Sources of Support/Disclosure

None of the authors have been supported by industry in this study, no founds were received, and they have no disclosure for this work.

## Figures and Tables

**Table 1:** 

Author	Epidemiology	Location	Mechanism	Germen	Treatment	Functional result	X-ray results
Keret et al., 1998 [2]	9-year-old male.	Left hand, 3rd metacarpal	Dog scratch	*Bartonella henselae*	Antibiotic therapy	Normal	Normal
Bickel et al., 1993 [3]	10 month old male.	Right hand, 3rd metacarpal	Hematogenous	*Coccidioides immitis*	Surgery + antibiotic therapy	Lost of followup at 3 months	Lost of followup at 3 months
Zitoun et al., 2003 [4]	6-year-old male.	Left hand, 4th metacarpal	Trauma	*Staphylococcus aureus*	Surgery + antibiotic therapy	Normal	Metatarsal shortening
Aebi and Ramilo, 1998 [5] case 1	5-year-old female.	Left hand, 3rd metacarpal	Chickenpox lesions	*Staphylococcus aureus*	Surgery + antibiotic therapy	Normal	Expansion and periosteal reaction
Aebi and Ramilo, 1998 [5] case 2	2-year-old male	Left hand, 3rd metacarpal	Chickenpox lesions	*Streptococcus pyogenes*	Surgery + antibiotic therapy	Normal	Normal
